# Sol–Gel Synthesis and Characterization of YSZ Nanofillers for Dental Cements at Different Temperatures

**DOI:** 10.3390/dj9110128

**Published:** 2021-10-29

**Authors:** Anastasia Beketova, Anna Theocharidou, Ioannis Tsamesidis, Athanasios E. Rigos, Georgia K. Pouroutzidou, Emmanouil-George C. Tzanakakis, Dimitra Kourtidou, Liliana Liverani, Marcela Arango Ospina, Antonios Anastasiou, Ioannis G. Tzoutzas, Eleana Kontonasaki

**Affiliations:** 1 Department of Prosthodontics, School of Dentistry, Faculty of Health Sciences, Aristotle University of Thessaloniki, 54124 Thessaloniki, Greece; anastasiabeketova@yahoo.com (A.B.); antheo@dent.auth.gr (A.T.); johntsame@gmail.com (I.T.); thanrigos@gmail.com (A.E.R.); 2 School of Physics, Aristotle University of Thessaloniki, 54124 Thessaloniki, Greece; gpourout@physics.auth.gr (G.K.P.); dikourti@physics.auth.gr (D.K.); 3 School of Dentistry, National and Kapodistrian University, 10679 Athens, Greece; tzanakak@dent.uoa.gr (E.-G.C.T.); ioannistzoutzas@gmail.com (I.G.T.); 4 Department of Materials Science and Engineering, Institute of Biomaterials, University of Erlangen-Nuremberg, 91058 Erlangen, Germany; liliana.liverani@fau.de (L.L.); marcela.arango@fau.de (M.A.O.); 5 Department of Chemical Engineering and Analytical Science, University of Manchester, Manchester M13AL, UK; antonios.anastasiou@manchester.ac.uk

**Keywords:** nanoparticle, chemical precipitation, yttrium, zirconium, gingival fibroblasts, toxicity tests, oxidative stress

## Abstract

Background: Yttria-stabilized zirconia nanoparticles can be applied as fillers to improve the mechanical and antibacterial properties of luting cement. The aim of this study was to synthesize yttria-stabilized zirconia nanoparticles by the sol–gel method and to investigate their composition, structure, morphology and biological properties. Methods: Nanopowders of ZrO_2_ 7 wt% Y_2_O_3_ (nY-ZrO) were synthesized by the sol–gel method and were sintered at three different temperatures: 800, 1000 and 1200 °C, and their composition, size and morphology were investigated. The biocompatibility was investigated with human gingival fibroblasts (hGFs), while reactive oxygen species (ROS) production was evaluated through fluorescence analysis. Results: All synthesized materials were composed of tetragonal zirconia, while nanopowders sintered at 800 °C and 1000 °C additionally contained 5 and 20 wt% of the cubic phase. By increasing the calcination temperature, the crystalline size of the nanoparticles increased from 12.1 nm for nY-ZrO800 to 47.2 nm for nY-ZrO1200. Nano-sized particles with good dispersion and low agglomeration were received. Cell culture studies with human gingival fibroblasts verified the nanopowders’ biocompatibility and their ROS scavenging activity. Conclusions: the obtained sol–gel derived nanopowders showed suitable properties to be potentially used as nanofillers for dental luting cement.

## 1. Introduction

Various nanoparticles have been proposed for the reinforcement of different dental materials such as restorative composite materials, glass ionomer cement, dental adhesives and calcium silicate cement. Among them, silica, alumina, glass and metal oxide nanoparticles led to improved commercial products with enhanced mechanical properties. In the last two decades, high purity zirconia (ZrO_2_) nanomaterials gained significant interest in dental technology, as they combine high toughness, high strength and high corrosion resistance with biocompatibility, lack of toxicity and antibacterial properties [1]. Although their primary application is in the fabrication of dense polycrystalline zirconia ceramics in the form of dental crowns, fixed partial dentures, implants, implant abutments, posts, orthodontic brackets, etc. [2,3], they have also been proposed as nano-filling materials [4,5] and nanocoatings [6,7], while due to their opaque white nature, they can also serve as radiopacity elements in cement [8] and root filling materials [9]. Zirconia filler nanoclusters with particle sizes ranging from 20 to 75 nm were used for strengthening commercial restorative dental composites such as Filtek Z250 and Harvard ZirkonCore [4]. The incorporation of ZrO_2_ nanoparticles in various matrices can result in composite materials with significantly improved flexural strength, fracture toughness and shear bond strength [10,11]. Due to their antibacterial properties, they could also be applied as fillers in various dental care materials such as toothpaste, lining materials and dental cement [12,13]. The reinforcement of resin luting cement by ZrO_2_-based nanofillers could be beneficial for the establishment of durable bonds with zirconia fixed restorations.

Zirconium oxide presents with three temperature-dependent crystal phases: monoclinic (m-ZrO_2_) is thermodynamically stable at room temperature and up to 1100 °C, tetragonal (t-ZrO_2_) between 1100 and 2370 °C and cubic (c-ZrO_2_), which is found at a higher temperature above 2370 °C [14,15]. Tetragonal zirconia presents with the most favorable mechanical properties due to the phenomenon of toughening transformation, i.e., its transformation under moisture and stress to the thermodynamically stable at room temperature monoclinic phase [16]. During the cooling of the material at 950 °C, conversion from the tetragonal to the monoclinic (t→m) form takes place [17]. This phase transformation is accompanied by a volume increase of ~4% [18]. A different transformation (c→t) can cause the creation of a different tetragonal phase (t’), which is rich in yttrium with a smaller crystal size and higher resistance to the t→m phase transformation [19]. In order to stabilize tetragonal zirconia at room temperature, various doping elements were tested, such as cerium, yttrium, calcium, magnesium, etc., and yttrium is the most utilized. Yttrium stabilized tetragonal zirconia (YSZ) has attracted worldwide interest as it presents high strength and toughness [20].

Various methods were proposed for the synthesis of zirconia nanopowder [21]. The most effective ones are wet-chemical synthesis approaches, such as sol–gel, co-precipitation and hydrothermal routes [22,23,24,25]. Using sol–gel synthesis, uniform, nano-sized powders with high purity can be produced [26]. This process is based on the hydrolysis and subsequent condensation reactions of inorganic salts and metal–organic compounds. These reactions lead to the formation of a sol which is converted into a gel. The gel is further processed with calcination at various temperatures to obtain a homogenous nanopowder. In the co-precipitation method, an aqueous solution is prepared where zirconia precursors are diluted, and then a chemical precipitant agent is added for the effective precipitation of metal hydroxides. The precipitated powder is subsequently rinsed, filtered and dried before calcination at various temperatures to receive the desired crystalline phases. The nucleation and growth mechanisms can be monitored by modifying the solution’s pH and temperature. It is an effective and low-cost method, although it generally results in a wide particle size distribution and agglomeration [27]. Hydrothermal routes usually involve water as the solvent and an initial co-precipitation at high temperatures and pressure in sealed containers to obtain a crystalline powder. It is also a low-cost and ecological method resulting in homogenous products, although presenting similar drawbacks of co-precipitation such as high agglomeration, which results in poor sinterability [28,29]. All of these methods necessitate precise control of all the involved parameters (pH, time, temperature, etc.) to receive the desired size and crystalline nature of nanoparticles. Nanoparticles with an average size below 50 nm were suggested as appropriate zirconia nanofillers in dental restorative composites and cement [13,30].

Despite the fact that pure monoclinic zirconia nanoparticles have been used as fillers in many dental materials [7], YSZ nanoparticles with tetragonal structure at room temperature have only scarcely been evaluated [31,32], although they may show higher enhancement of the mechanical properties of dental composites and cement. The aim of this study was to synthesize yttria-stabilized zirconia (YSZ) nanopowders, to be used as nanofillers in dental cement by the sol–gel method and to investigate the impact of different sintering temperatures on their crystal structure, morphology and biocompatibility. The null hypothesis was that sintering temperature would not affect the biocompatibility of the synthesized materials.

## 2. Materials and Methods

### 2.1. Synthesis of Nanoparticles

ZrO_2_ 7 wt% Y_2_O_3_ nanoparticles were synthesized by the sol–gel method using zirconium oxychloride octahydrate (ZrOCl_2_ 8H_2_O) and yttrium nitrate hexahydrate (Y(NO_3_)_3_ 6H_2_O) as starting materials [33,34]. Raw materials were dissolved in double distilled water, mixed and then an aqueous solution of ethylene glycol and an aqueous citric acid concentrate was added under heating and stirring. The molar ratios of citric acid:metal and citric acid:ethylene glycol were 3.65 and 1, respectively. The materials were heated stepwise to the temperatures of 100 °C, 200 °C and 300 °C for 3 h/each to eliminate organic materials [33]. The obtained gel was sintered at three different temperatures: 800, 1000 and 1200 °C for 2 hours after differential thermal and thermogravimetric analyses (DTA/TG). The obtained calcinated materials were ground in a mortar into fine powders (nY-ZrO800, nY-ZrO1000, nY-ZrO1200).

### 2.2. Differential Thermal and Thermogravimetric Analysis (TG-DSC) 

The thermal behavior of the dried gel was investigated by Thermogravimetric Analysis and Differential Scanning Calorimetry (TG-DSC) performed in dry air from room temperature up to 1300 °C, with a heating rate of 10 °C min^−1^ (SETSYS 16/18, SETARAM, Lyon, France). The sample (≈35 mg) was placed in alumina crucibles while an empty alumina crucible was used as a reference.

### 2.3. Fourier Transform Infrared Analysis (FTIR)

The FTIR transmittance spectra of the nanoparticles sintering were obtained by the KBr technique. A Spectrometer (Spectrum 1000, PerkinElmer, Inc., Waltham, MA, USA) was employed measuring in the MIR region (4000–400 cm^−1^) with a resolution of 4 cm^−1^ and performing 32 scans per spectrum.

### 2.4. X-Ray Diffraction Analysis (XRD)

The XRD analysis of the nanoparticles was performed using a diffractometer (Rigaku Ultima, Rigaku, Japan) with Ni-filtered CuKa radiation (λ = 0.1542 Å). A 2θ range of 5–90° was measured, with a scanning speed of 0.05 ° 2θ/s, 1.5 s per step. The ICDD PDF-4+ (2009) database was used for the identification of the phases contained in the studied samples. The crystallite size was calculated with the Scherer’s formula: D = 0.89λ/β cosθ, where D is the crystallite size (nm), λ is the wavelength of X-ray beam (nm), β is the full width at the half maximum of the main peak, and θ is the diffraction angle (°). Rietveld analysis of XRD patterns was performed to quantify the crystalline phase contents. 

### 2.5. Scanning Electron Microscopy-Energy-Dispersive X-ray Spectroscopy (SEM/EDX) 

The morphology of all samples was determined by Scanning Electron Microscopy SEM (Auriga Base, Carl-Zeiss) associated with an energy dispersive X-ray (EDX) analyzer (X-Max^N^, Oxford Instruments, Abingdon, UK) to detect their chemical composition during SEM observation. 

### 2.6. Transmission Electron Microscopy (TEM) 

For TEM imaging, the NPs samples were dispersed in ethanol and placed in an ultrasonic bath for 10 min. Then, a drop of the suspension was placed onto a Lacey Carbon Film (Agar Scientific Ltd., Stansted, UK). For imaging and morphology analysis of nanoparticles, a latest-generation Field Emission Gun Transmission Electron Microscope (Talos F200X) was utilized. The mean particle size of the samples was determined by measuring the size of 50 particles. The exact distance from the central spot and each one of the diffracted spots was measured in program Image J. Experimental interplanar spacing (d) was estimated from the formula: d_spacing_ = λL/R [35,36].

### 2.7. Dynamic Light Scattering Analysis (DLS)

Dynamic light scattering (Zetasizer Nano ZS) was used for the estimation of hydrodynamic size and polydispersity index (PDI) of the nanoparticles. Measurements were performed at 25 °C after 5 min sonication of the samples that contained the different nanoparticles dispersed in distilled water at a concentration of 1 g/L.

### 2.8. Establishment of Primary Cell Cultures

Human gingival fibroblasts were grown in primary culture from free gingiva biopsies received from young, healthy donors after extraction of their third molars. The Institutional Ethical Committee approved the protocol (#35/07-05-2018), and volunteer patients signed their informed consent forms before extractions. In brief, gingival tissue biopsies were placed in 25 cm^2^ culture flasks with Dulbecco modified Eagle medium (DMEM, Invitrogen) supplemented with 10% Fetal Bovine Serum (FBS, Invitrogen) and penicillin (100 units/mL), streptomycin (100 mg/mL) and Amphotericin B (0.25 mg/mL) to allow the outgrowth of cells. The flasks were incubated at 37 °C in a humidified atmosphere with 5% CO_2_, and the medium was replaced twice a week. When the primary cell culture reached confluence (70–80% of the flask), cells were detached with trypsin, transferred to a larger 75 cm^2^ flask (passage 1) and then subcultured for further experiments. Cells from the 5th and 6th passages were used for all experiments.

### 2.9. Evaluation of Cell Viability by the MTT Assay

Mitochondrial activity evaluation of human gingival fibroblasts with nY-ZrO was performed using the MTT assay (3-(4,5-dimethylthiazol-2-yl)-2,5-diphenyltetrazolium bromide). Cells were seeded in 96 well-plates (3 × 10^4^ cells/well) and left 24 h to attach in a 5% CO_2_ incubator at 37 °C. A stock solution of 2 mg of NPs per 1mL of culture medium was prepared, and a series of dilutions with nanoparticles at different concentrations (0.1, 0.25 and 0.5  mg/mL) were evaluated after 24 and 72 h of incubation. Those concentrations were selected based on previous studies on the toxicity of various nanoparticles [37,38,39]. Untreated cells served as a positive control, while cells cultured with a culture medium without fetal bovine serum served as a negative control. The proliferation of the cells was calculated by measuring the mitochondrial dehydrogenase activity of living cells, which was verified by the transformation of the yellow tetrazolium salt into blue formazan crystals by using dimethyl-sulfoxide (DMSO) (Sigma Aldrich, St. Louis, MO, USA) as dissolvent. Optical density was determined spectrophotometrically by a microplate reader (Epock, Biotek, Biotek Instruments, Inc, Winooski, VT, USA). Measurements were performed at a wavelength of 545 nm with a reference filter at 630 nm. Experiments were performed in triplicates. The results were expressed as % percentage of the control average optical density value.

### 2.10. Fluorescence Analysis for the Detection of ROS Levels

The cell-permeable ROS-sensitive probe 2′,7′-dichlorodihydrofluorescein diacetate (H2DCFDA) was used for the detection of reactive oxygen species [40], which fluoresces at 520 nm (λex480 nm) upon oxidation. A stock solution of 0.5 mM H2DCFDA in DMSO was prepared. In order to avoid autofluorescence, controls were prepared without H2DCFDA but only with its solvent, DMSO. The probe was added to the well plates of human gingival fibroblasts treated at different concentrations of yttria-stabilized zirconia nanofillers for 24 and 72 h and left to incubate for 30 min (oxidation). Measurement of the fluorescence was performed after transferring the desired suspensions in 96-well black microplates using a SAFAS Xenius fluorometer. The relative fluorescence is expressed as “% maximal emission” as determined with the software “Xenius”.

### 2.11. Statistical Analysis

Statistical analysis of the cell viability and ROS levels data was performed with the Paired Samples *t*-Test using the SPSS software. The level of statistical significance was set at 0.05 (*p* < 0.05).

## 3. Results

### 3.1. TG-DSC Analysis

**Figure 1:**
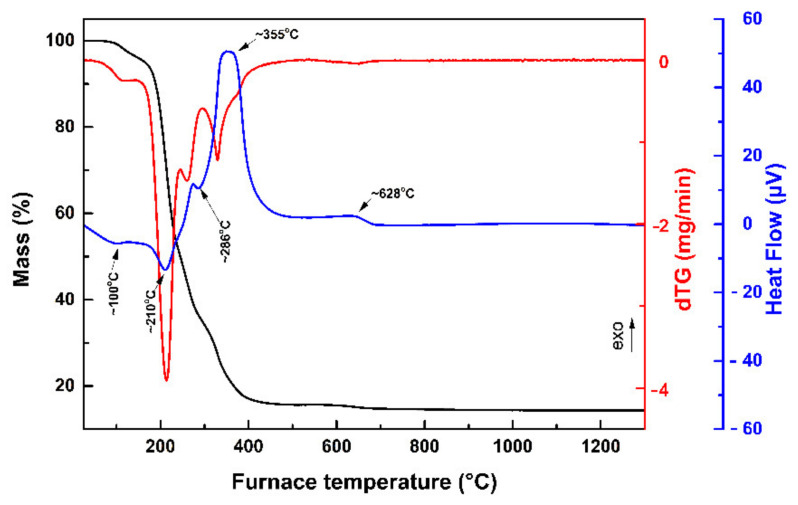
TG, dTG and Heat Flow curves of the gel at 10 °C/min heating rate in dry air.

The mass loss along with its first derivative (dTG) and the heat flow curves of the prepared gel is presented in Figure 1.

From the dTG curve, it can be concluded that the overall weight loss (85.3%) occurred in five main stages. The first step took place at temperatures between 26 and 150 °C, accompanied by an endothermic peak at ~100 °C, and is attributed to the evaporation of the absorbed water (3.5% mass loss) [33,34,41,42]. In the temperature range of 150–240 °C, the second step of the degradation presented an endothermic peak at ~210 °C that can be ascribed to the release of nitrates and/or the citrate matrix’s decomposition (45% mass loss) [43,44]. The third stage of the gel’s degradation occurred around 240–300 °C with an endothermic peak appearing at ~286 °C, possibly due to the decomposition of zirconyl oxalate (ZrOC_2_O_4_) and Y_2_(C_2_O_4_)_3_ (20.9% mass loss) [43,45]. An exothermic peak at ~355 °C took place on the fourth step of the process, which took place at temperatures between 300 and 540 °C. This peak could be attributed to the degradation of organic and organometallic compounds, CO_2_ removal and the destruction of the polymeric network [34] or to the oxidation of the residual organic compounds [44] (mass loss 15.9%). During the final step of the degradation that occurred in the temperature range of 540–900 °C, an exothermic peak appeared at ~628 °C, possibly related to the removal of the element carbon from the final compound [44] and/or a crystalline–amorphous phase transition [33,45].

### 3.2. FTIR Analysis

**Figure 2:**
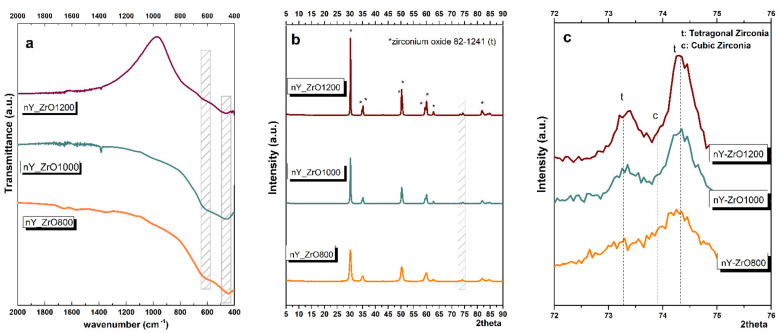
(**a**) FTIR; (**b**) XRD analysis of the zirconia nanoparticles sintered at 800, 1000 and 1200 °C, respectively. Higher magnification of XRD patterns in the area of 72–76 2θ is presented in (**c**) to unravel the splitting of the peaks due to the presence of the tetragonal phase.

Infrared vibrational spectra of Y-Zr powders, sintered at temperatures 800, 1000 and 1200 °C, are presented in Figure 2. FTIR transmission spectra of all the specimens showed broad bands at about 430–440 cm^−^^1^ and a weak broad band at around 600–650cm^−^^1^ characteristic for the tetragonal phase [46]. Apparently, no monoclinic phase was detected in none of the specimens because of the absence of the distinctive sharp band for this phase at 740cm^−^^1^. Low-intensity peaks at 1400 and 1650 cm^−^^1^ of nY-ZrO800 specimen might be attributed to the symmetric and asymmetric stretching vibrations of the carboxylate groups of organic components of decomposed gel [34].

### 3.3. XRD Analysis

The XRD patterns of the three experimental materials are displayed in Figure 2, while the percentages of crystalline phases are presented in Table 1. The diffraction peaks of all nY-ZrO1200 specimen show the formation of pure tetragonal zirconium yttrium oxide Zr0.9Y0.101.95 without any detectable secondary phases. The distinguishing characteristic peaks for tetragonal zirconia phase occur at 2θ = 30 to 45 for the (111), (002), (200), (202) and (112); at 2θ = 45 to 65 for the (202), (220), (113), (311) and (222); and at 2θ = 72 to 85 for (004), (400), (331), (313), (420) and (402) reflections [47,48]. It is important to mention that the double peak around 73.2 and 74 2θ angles are characteristic for tetragonal zirconia crystal [48,49]. Specimens nY-ZrO1000 and nY-ZrO800 also contain ~5% and ~20% of cubic zirconium yttrium oxide, respectively. By increasing the sintering temperature from 800 to 1000 and 1200 °C, the peaks appeared sharper and more intense. At the same time, the full width at half maximum (FWHM) of diffraction pattern became narrower, indicating increased crystallinity of the specimens. Thus, the crystallite size of YSZ nanoparticles calculated by the Sherrer equation was 12.1nm for nY-ZrO800, 29.2nm for nY-ZrO1000 powder and 47.2nm for nY-ZrO1200.

### 3.4. SEM/EDX

**Figure 3:**
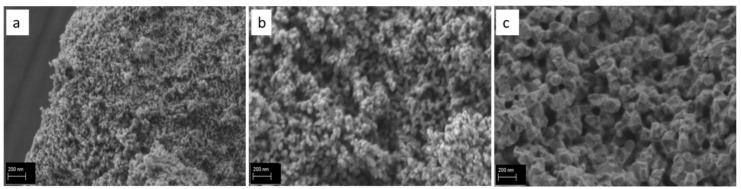
SEM images of the nY-ZrO powders sintered at 800 ((**a**)-nY-ZrO800), 1000 ((**b**)-nY-ZrO1000) and 1200 °C ((**c**)-nY-ZrO1200), showing increase in crystalline size with increase in sintering temperature.

SEM analysis (Figure 3) in high magnifications (100,000×) showed the fine submicron-sized (~10–15 nm) particles with uniform spherical morphology in nY-ZrO800 specimens. With the increase in sintering temperature to 1000 °C, grains become bigger in size (30–50 nm) and have a low agglomerated microstructure. Finally, specimens sintered at 1200 °C seem more agglomerated and have polyhedral morphology with particle sizes of more than 100 nm. EDX analysis of three specimens detected Zr, Y and minor traces of Hf, with a Y/Zr ratio around 0.06–0.07.

### 3.5. TEM

**Figure 4:**
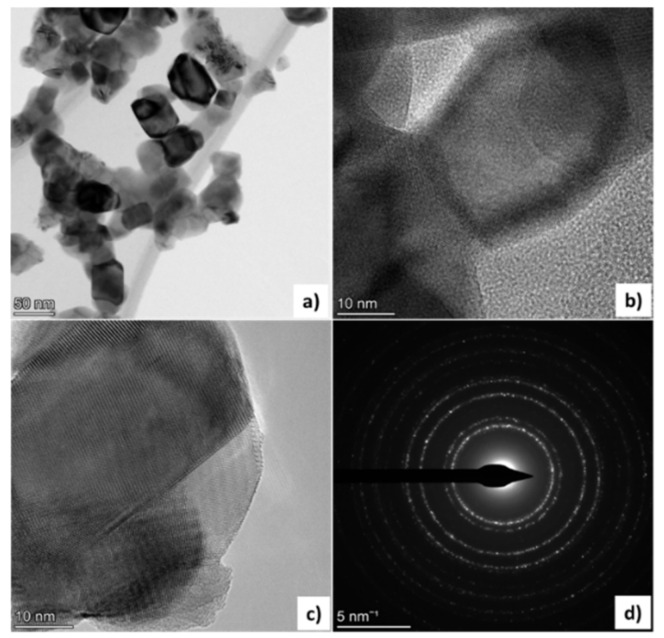
TEM image showing morphology of nanoparticles sintered at 1000 °C (**a**–**c**), and electron diffraction pattern (**d**).

The obtained TEM image of nY-ZrO powders sintered at 1000 °C is shown in Figure 4a. The observed nanoparticles are uniform, homogeneously distributed and well defined. Their morphology is polyhedral, and their size ranges between 9 and 53 nm, with an average calculated particle size of 28.4 nm (±11 nm). In images with higher resolution (Figure 4b), crystal lattice could be observed, while dark inclusions indicate well-crystallized grains. The electron pattern of crystals corresponds to pure tetragonal zirconia, which can be estimated from calculations of interplanar spacings. (Table 2).

### 3.6. DLS

The average size of all synthesized powders, analyzed by Dynamic Light Scattering techniques (DLS), were estimated 243.9 nm for nY-ZrO800, 188.96 for nY-ZrO1000 and 364.00 for nY-ZrO1200 (Table 3). The low polydispersity index, especially in the nY-ZrO 800 and nY-ZrO1000 samples (<0.28), indicates narrow size distribution of grains and good quality of the obtained materials.

### 3.7. Evaluation of Cell Viability

**Figure 5:**
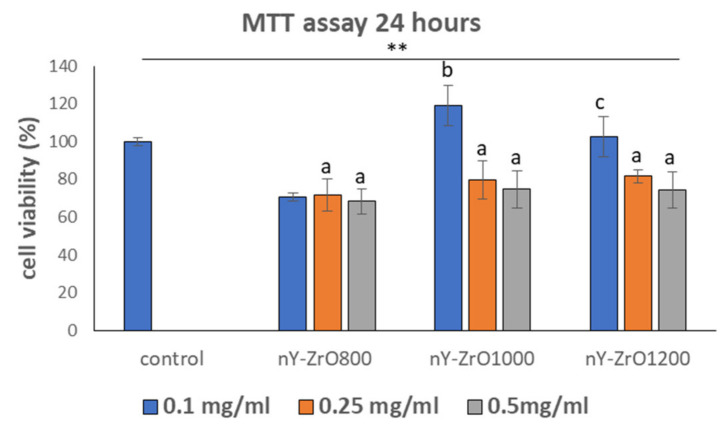

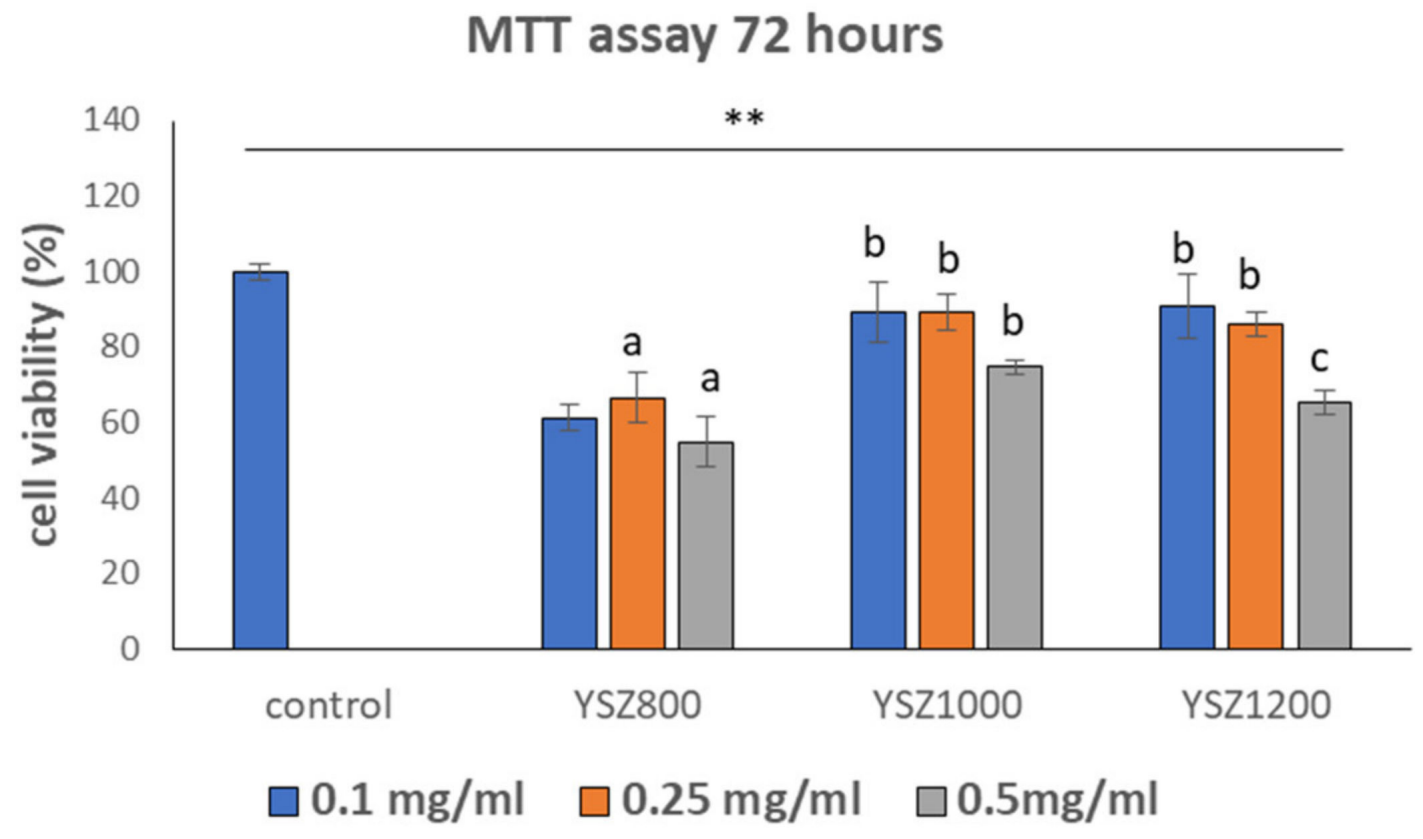
MTT results of cell viability at different concentrations (0.1, 0.25, 0.5 mg/mL) of yttria stabilized zirconia nanofillers. ** Indicates statistically significant difference (*p* < 0.001) between treated and untreated cells without nanoparticles (control), while different letters suggest statistically significant differences (*p* < 0.001) among concentrations.

MTT assay of the tested specimens (Figure 5) showed that nY-ZrO1000 and nY-ZrO1200 presented a biocompatible biological behavior as compared to the positive control in 24 and 72 h (*p* < 0.001). The lowest cell viability/proliferation was found in the highest concentrations of nanoparticles (0.5 mg/mL) in all the experimental groups (*p* < 0.001). The most pronounced and statistically significant increase in cell viability (*p* < 0.001) was reported for cells treated with 0.1 mg/mL of nY-ZrO1000 after 24 h of incubation. The lowest optical density values were recorded in the nY-ZrO800 group at 72 h, suggesting its mildly toxic behavior. Time dependence and dose dependence were not observed for the experimental dose and time points, except for cells treated with nY-ZrO1200 after 72 h of incubation, presenting a statistically significant dose-dependent behavior (*p* < 0.05).

### 3.8. Fluorescence Analysis for the Detection of Reactive Oxygen Species Levels

**Figure 6:**
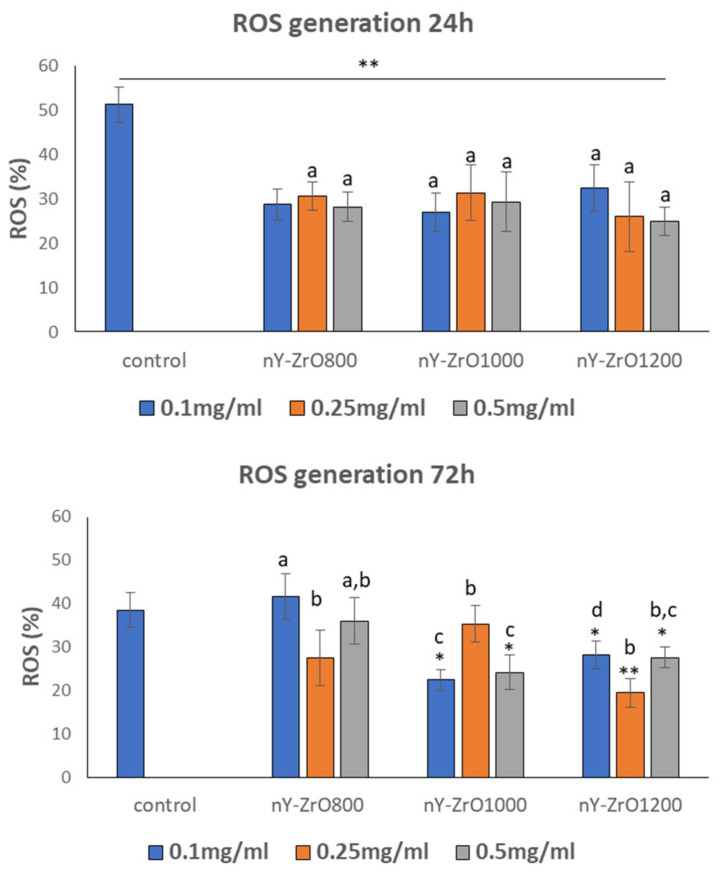
Reactive oxygen species (ROS) measured using H2DCFDA in HGF cells treated for 24 and 72 h at different concentrations (0.1, 0.25, 0.5 mg/mL) of yttria-stabilized zirconia nanofillers. * Indicates statistically significant difference (*p* < 0.05) between treated and untreated cells without nanoparticles (control), ** indicates statistically significant difference (*p* < 0.0001) between treated and untreated cells without nanoparticles, while different letters suggest statistically significant differences (*p* < 0.001) among concentrations.

Figure 6 illustrates that after 24 h of incubation, all specimens showed a statistically significant reduction of reactive oxygen species as compared to the control (*p* < 0.001). After 72 h, in nY-ZrO1000 and nY-ZrO1200 specimens, a significant reduction in reactive oxygen species levels was observed, which was not dependent on NP concentrations (*p* < 0.01). Higher antioxidant activity of YSZ800 nanoparticles was observed at the 72h time point in the concentration of 0.25 mg/mL, indicating its antioxidant properties.

## 4. Discussion

Because of the favorable properties of zirconia nanoparticles, their synthesis has attracted wide scientific interest over the years, and various methodologies were applied to optimize their size, crystalline state and morphology, depending on the targeted application [7]. ZrO_2_ and particularly YSZ nanoparticles present high ionic conductivity, mechanical strength, chemical inertness, high melting point, low thermal conductivity [50] as well as biocompatibility [51], characteristics that make zirconia nanoparticles attractive for a wide range of applications. Yttria-stabilized zirconia nanoparticles offer multiple advantages compared to pure ZrO_2_ in terms of high mechanical properties, antibacterial properties and low thermal conductivity that make them attractive candidates for fillers in dental cement and composite materials [52,53].

In the present study, YSZ nanoparticles were synthesized with the sol–gel technique based on the Pechini method, as described previously by Hajizadeh-Oghaz [24]. In their study, spherical tetragonal nanoparticles were synthesized with an average size of 29 nm, as determined by Scherrer’s formula. This size corresponds to calcination at 1000 °C and is in close agreement with the results of the present study (28.4 nm). Calcination temperature was found to be the most important factor influencing particle size of YSZ, as also pointed out in similar studies [24,33,54]. For example, Maridurai et al. [54] synthesized YSZ nanoparticles by the co-precipitation method and observed an average particle size of 17 nm with TEM analysis and predominantly spherical shape after calcination at 700 °C. Although a different technique was used for the synthesis, Scherrer’s equation indicated an average size of 12 nm [54], similar to the size of nanoparticles in the present study after calcination at 800 °C. However, in the study of Maridurai et al. [54], cubic zirconia was the predominant crystalline phase and not the tetragonal one, as in the present study.

Apart from yttrium, other elements were used to stabilize tetragonal ZrO_2_ nanoparticles, such as ytterbium (Yb) and gadolinium (Gd) [55]. With these elements, XRD revealed an increase in size with temperature, as an average size of 19 nm was calculated when calcination was performed at 600 °C and 43 nm following calcination at 1000 °C [55]. This finding was also observed in the present study and is justified by the temperature-induced grain growth and densification. As shown by SEM micrographs in Figure 3, heating at 1200 °C led to significant consolidation of the particles.

Khajavi et al. [56] recently synthesized and evaluated Ce-Y co-doped zirconia nanoparticles by the hydrothermal method, which presented a spherical shape and average size of 8.6 nm after calcination at 900 °C. In addition, the authors reported that a predominantly tetragonal phase of zirconia was detected [56], which is in agreement with the present study, although their nanoparticles presented a smaller average size. They also emphasized the significance of retaining the tetragonal phase after heat treatment, which is correlated to mechanical properties enhancement through the transformation toughening mechanism. In the present study, the tetragonal phase is well retained at all temperatures, as verified by the double peaks of the (002)/(200), (113)/(311), (400)/(004) and (402)/(420) planes in the XRD pattern of the nY-ZrO1000 and TEM analysis of the nY-ZrO1000 [48,49]. The broadening of the respective peaks in the pattern of the nY-ZrO800 suggests peak overlapping [48], which is a common finding, as in X-ray diffraction analysis, the cubic and tetragonal structures cannot be easily distinguished [48]. A high amount of tetragonal zirconia and no monoclinic phase were found at 800 °C, quite below the phase equilibrium (1175 °C), which can be justified by the nano-scale size of powders that present high surface area. After the studies of Garvie [57], which suggested that 30 nm of crystalline size is the critical factor for the stabilization of the tetragonal phase at lower temperatures, many studies suggested that an even smaller size (18 nm [58] or 10 nm [59]) of zirconia nanocrystals do not inhibit its stabilization, depending on various parameters such as interfacial energy, strain energy, hydrostatic pressure, water vapor, ion doping, oxygen vacancies, etc. [60]. Therefore, these critical size factors are strictly applicable only for strain-free ZrO_2_ nanocrystallites of spherical or near-spherical shape in contact with air at ambient pressure and temperature. In the present study, the average size calculated by XRD was below 30 nm at temperatures below 1000 °C, which corroborates well with the proposed theories [60]. Similarly, Tailor et al. [34] synthesized YSZ nano-clusters for thermal insulation by the sol–gel method and, in agreement with our study, mainly detected the tetragonal phase of YSZ and average particle size of around 40 nm. In the present study, a very small amount of cubic phase was calculated by the Rietveld method (<5%) for the nYZ-1000, which was reported in cases of ultrafine crystallites, in the range of 2–20 nm [61,62] and in doped YSZ nanoparticles [56].

Nanoparticles are prone to spontaneous agglomeration due to high specific surface area. This surface area is responsible for the lowering of the sintering temperature of nanoparticles compared to the sintering temperature of their micro-sized counterparts [63]. It was shown that the activation energy for densification of ZrO_2_ nanoceramics is 50% less than that of submicron ZrO_2_ [64]. Stolzenburg et al. [65], using a solvothermal synthesis method, observed ZrO_2_ nanoparticles agglomerates of size around 200 nm and justified their results by a probable instantaneous self-arrangement of nanoparticles after nucleating in the tetragonal phase. In the present study, the respective values were 243.9 nm, 188.96 nm and 364.00 nm, following heating at 800, 1000 and 1200 °C, respectively. At high temperatures, particles draw near and neck-shape junctions between adjacent nanoparticles permit densification and pore shrinkage, as in the case of nY-ZrO1200. DLS measurements in the present study suggest a moderate to low polydispersity in all samples, as the polydispersity index (PDI) values are below or around 0.3, but the high average nanoparticle diameter calculated may be attributed to the tendency of nanoparticles to assemble in nanoclusters or to the severe distortion of DLS measurements due to nanoparticles polyhedral morphology [66,67]. Considerably larger nanoparticles sizes compared to TEM and SAXS were recorded by DLS [68], and even slight soft agglomeration or a small percentage of larger particles can significantly increase the DLS calculated particle size distribution [66,67].

In order to investigate the biocompatibility and antioxidant activity of the experimentally obtained yttria-zirconia nanoparticles, MTT assay and fluorescence analysis for detection of ROS levels were performed. Nanoparticles sintered at higher temperatures of 1000 and 1200 °C showed biocompatible behavior and thus are not expected to cause any adverse effects after their addition to luting cement. Nanoparticles sintered at 800 °C presented mildly toxic behavior, probably due to finer particle size as evidenced from the SEM microphotograph (Figure 3a). Nanoparticles of small size <10 nm can either penetrate the cell membrane or enter the nucleus, thus being mildly or heavily toxic [69]. Pan et al. [70] reported that gold NPs of 15 nm in size were 60 times less toxic than 1.4 nm NPs for fibroblasts, epithelial cells, macrophages and melanoma cells. In agreement with our study, Zhang et al. [71] reported in vitro cytocompatibility of ZrO_2_ nanopowders (<50 nm) with MG-63 cells and L929 fibroblasts in a dose-dependent manner. Cell culture studies with human umbilical vein endothelial cells (HUVECs) cultured with 500 μg/mL (4.06 mM) of ZrO_2_ nanoparticles (30–40 nm) also showed relatively high cell viability (>80%) [72]. In a study by Rutherford et al. [73], YSZ nanoparticles (approximately 7 nm) with different molar concentrations of yttria and nano-sized ZrO_2_ showed biocompatible behavior with bone marrow mesenchymal stem cells. Even at a high concentration of nanoparticles, cells maintained their morphology. Further research is needed to elucidate the underlying factors and mechanisms affecting zirconia nanoparticles’ cytotoxicity at different cell lines. In general, immediately after nanoparticles’ contact with the biological environment, a certain layer of proteins will adsorb on their surface, guiding further cells behavior. Applying shorter time points in the MTT test (i.e., 1, 3 or 6 h) [74] could be an effective strategy to elucidate the initial effect of these nanoparticles in cell attachment. However, despite the initial levels of cell attachment, the main effect of nanoparticles on cells’ viability is already observed at the first 24 h, with a decrease in an increase in cells’ proliferation, but after 48 or 72 h, usually, a balance is acquired if the material is biocompatible. Therefore, we employed a 3-day MTT test as indicative of the materials’ toxicity, although shorter or longer evaluation times would be more informative. In addition, further tests are needed to provide information on the mechanisms of cell damage or death and to explain the decreased biocompatibility of the nY-ZrO800 observed in this study. Despite the scientific knowledge related to cell death mechanisms, further work is needed to elucidate the toxicological behavior of the various nanomaterials and to clarify the particular factors that affect cell fate in their presence. Moreover, there is a need to evaluate if this biological behavior can affect cell viability when these nanoparticles are used as fillers in dental cement or adhesives. In this respect, in-depth analysis, as presented in the study of Pagano et al. [74], and the use of direct cytotoxicity tests on dental cement, adhesives or composites with nano zirconia nanoparticles fillers should be performed.

The antioxidant potential is an important characteristic of nanoparticles specifying their free oxygen radical scavenging activity and their ability to protect cells from oxidative damage. In the current study, all specimens showed a statistically significant reduction in ROS, indicating the free radical scavenging activity in 24 h, irrespectively of the NPs concentrations added. It seems that the tested nanofillers were effective in neutralizing oxidative stress by stabilizing a basal production of ROS. Recently, the pronounced scavenging activity of ZrO_2_–CeO_2_ nanoparticles attributed to their oxygen storage capacity was reported by Tsai et al. [75]. Another study found the enhanced antioxidant activity of Fe_3_O_4_ stabilized zirconia nanoparticles [76]. At the same time, several studies reported that non-stabilized ZrO_2_ nanoparticles could generate ROS and damage the cellular components as well as the DNA [38,77]. Although a ROS scavenging mechanism due to the presence of oxygen vacancies in nanomaterials can contribute to those results [78], in the study by Alzahrani et al. [79], dose-dependent apoptosis and genotoxicity of YSZ nanoparticles in human skin keratinocyte cells were correlated with an increase in reactive oxygen species production at concentrations higher compared with the present study (up to 60 µg/mL), suggesting that maybe other mechanisms control the overall ROS production and/or scavenging. Such diversity in results could probably be attributed to differences in applied protocols and cell lines, as well as the chemical reactivity of pure zirconia as compared to its yttria-stabilized form.

Many studies used pure zirconia nanoparticles to enhance the mechanical properties of dental restorative composites, to enhance the bond strength to dentin of dental cement and adhesive systems or to increase the radiopacity of dental adhesives (Table 4). A recent review outlined the beneficial role of the incorporation of nanocompounds in dental materials with regards to the promotion of their bond’s stability [80].

Spherical YSZ nanoparticles of size ranging from 20 to 50 nm were previously produced by laser vaporization and incorporated in dental adhesives [32]. Zirconia nanoparticle incorporation into the primer or adhesive resin solution of a multi-purpose adhesive system increased the bond strength significantly to dentin, and even higher strength was achieved when increased amounts were incorporated into the primer. Martins et al. [95] incorporated pre-fabricated, commercially available ZrO_2_ nanoparticles (20–30 nm) in dental adhesives and found increased radiopacity and micro-hardness.

It was also proposed that ZrO_2_ nanoparticles can be incorporated in resin-based composite materials after appropriate silanization [96]. In the study by Kaizer et al. [96], the average particle size incorporated was 37.3 nm, and it increased to 81.2 nm following their silica coating. Nanohybrid resin composites with 40 nm zirconia nanoparticles presented improved and more stable physical properties compared with commercial dental composites or composites reinforced with nanosilica [97]. In a recent study, zirconia nano-fillers were incorporated in bis-GMA composite resins [98] up to 5–20 wt%. SEM images indicated spherical particles with sizes ranging between 20 and 50 nm, and a significant increase in bending strength was recorded for the composites.

Experimentally reinforced glass ionomer cement has been described in the literature recently. Gjorgievska et al. [85] attempted the incorporation of ZrO_2_ nanoparticles of average size 80 nm in glass ionomer restorative cement and reported fewer air voids in all nanoparticle-containing cement, which resulted in fewer cracks within the matrix of the cement, increasing their strength. Laiteerapong et al. [99] manufactured restorative glass ionomer cement after incorporation of pre-fabricated zirconia nanoparticles of size under 100nm (ZrO_2_) and investigated the genotoxicity of their eluates on human gingival fibroblasts. They used nano and micro-sized zirconia particles up to 10% *w*/*w* and concluded that zirconia modified GICs had no genotoxic effect on HGFs in vitro. Sajjad et al. [82] synthesized nano ZrO_2_–SiO_2_–HA, which was incorporated in Fuji IX GIC restorative material and detected particles comprising of spherical ZrO_2_ and SiO_2_ crystals and rod shape HA crystals.

Further studies showed that dental materials reinforced with ZrO_2_ nanoparticles present cell proliferating and antimicrobial properties. Specifically, Silva et al. [100] used ZrO_2_ nanoparticles to reinforce a calcium silicate-based cement and observed an increase in fibroblast proliferation and reduced duration of the inflammatory response by rat fibroblasts. In the study by Bosso-Martelo et al. [101], 74 nm-sized ZrO_2_ were incorporated into calcium silicate-based cement, resulting in bioactive materials, as evidenced by the hydroxyapatite precipitates on the surface of the specimens. In restorative glass ionomer cement, relative biocompatibility to human gingival fibroblasts was reported by Laiteerapong et al. [99]. Antimicrobial effects against microorganisms found in the oral cavity, particularly against Gram-negative bacteria, were reported by Fathima et al. [12]. In their study, particles synthesized by a method involving precipitation presented irregular spherical or spherical shapes, and their size ranged between 15 and 21 nm. The findings above indicate that the applied synthesis method in the present study led to smaller YSZ particle sizes (10–15 nm and 30–50 nm, respectively, for sintering temperatures of 800 °C and 1000 °C) when comparing them to the ZrO_2_ nanoparticles used in most studies.

It can be assumed that the favorable biological properties and crystallographic characteristics observed in the present study could allow applications of these nanoparticles in dental materials that require strict working times, viscosity and film thickness, such as luting cement. Other applications could include restorative materials as well as dental adhesives and root-end filling materials. The existing evidence on their biological and mechanical properties is promising with regards to their use as optimized fillers. However, this study has some limitations. A more detailed analysis should be performed in terms of explaining the underlying mechanisms of cells response and the differences depending on the sintering temperature. A more detailed TEM analysis on the nY-ZrO800 and nY-ZrO1200 should provide clarifying information on the role of any particular structural and morphological characteristics of nanoparticles on their biological response and ROS production to allow optimization of their production. Their biocompatibility should also be evaluated in comparison with pure ZrO_2_ nanoparticles to elucidate any potential effect of yttrium in their composition. Future studies in light of the above and the use of other cell lines such as dental pulp stem cells should be considered for conclusive results.

In the present study, yttrium stabilized zirconia nanoparticles were synthesized through a sol–gel-based method, and their biocompatibility were evaluated after sintering at various temperatures. As a different biological behavior was observed depending on sintering temperature, the null hypothesis was rejected.

## 5. Conclusions

Pure tetragonal YSZ nanopowders with low agglomeration were successfully synthesized by the sol–gel method at different temperatures. The size and crystallographic characteristics of the synthesized nanoparticles suggest the heat treatment at temperatures ≤1000 °C can lead to optimum properties, making YSZ nanoparticles potentially suitable as nanofillers for resin luting cement in dentistry. The results of the present study suggest that the sol–gel method is an effective alternative to traditional high-temperature synthesis techniques for the stabilization of the tetragonal zirconia at room temperature and the elimination of any monoclinic traces.

## Figures and Tables

**Table 1:** Content of tetragonal (t) and cubic (c) crystalline phases in wt% sintered specimens.

Specimen	t	c
nY-ZrO800	79.65%	20.35%
nY-ZrO1000	95.13%	4.87%
nY-ZrO1200	100.00%	0%

**Table 2:** Experimental and theoretical *d*-value (interplanar spacing) of the phase identified as tetragonal Zirconia (Identification Card Number# 82-1241).

Ring	Diam. cm	R cm	d_spac_ A°	ICDD #82-1241	St.Dev.	St.Dev %	hkl
Ring 1	6.5	3.250	2.892	2.963	0.0239	2.3924	101
Ring 2	7.6	3.800	2.474	2.559	0.0332	3.3151	110
Ring 3	10.3	5.150	1.825	1.818	0.0043	0.4260	112
Ring 4	12.45	6.225	1.510	1.544	0.0221	2.2058	103
Ring 5	13	6.500	1.446	1.482	0.0239	2.3924	202
Ring 6	15.65	7.825	1.201	1.212	0.0085	0.8503	104
Ring 7	17.35	8.675	1.084	1.144	0.0530	5.2986	310

**Table 3:** Results from DLS analysis.

Specimen	Size (d.nm)	Standard Deviation	%Std Deviation	Pdl
nY-ZrO800	243.890	2.345	0.961	0.265
nY-ZrO1000	188.956	5.831	3.086	0.277
nY-ZrO1200	364.003	8.216	2.257	0.376

**Table 4:** Studies involving zirconia nanoparticles in dental cement, composites and adhesives.

Authors	Zirconia Nanoparticles Type/Size (nm)	Amount of Filler (%) w.t.	Evaluated Property	Main Results
Dental Cements				
Gjorgievska et al. [81]	ZrO_2 (_80 nm) * Glass Ionomer Cement	2, 5, 10	-compressive strength element release profile	Increased compressive strength, no release of Al, Zr or Ti
Sajjad et al. [82]	ZrO_2 (_114 nm) * Glass Ionomer Cement	3, 5, 7, 9	-compressive strength -flexural strength -surface roughness	Increase in compressive and flexural strength
Alobiedy et al. [83]	ZrO_2 (_20 nm) * Glass Ionomer Cement	3, 5, 7	-compressive strength -micro-hardness -biaxial flexural strength wear rate loss	Favorable effect on biaxial flexural strength, micro-hardness, wear rate loss
Ab Rahman et al. [84]	ZrO_2_ (40 nm) * Glass Ionomer Cement	1, 3, 5, 7, 9, 15, 20	-hardness color	Increased hardness and aesthetics
Gjorgievska et al. [85]	ZrO_2_ (80 nm) * Glass Ionomer Cement	10	-compressive strength	Increased compressive strength
Li et al. [86]	ZrO_2 (_200 nm) * Tricalcium Cement	5, 10, 20, 30, 50	-mini-fracture toughness -bioactivity -cytotoxicity	Increase in biocompatibility
Rahimi et al. [87]	ZrO_2_ (<100 nm) * Portland cement	30	-viability of human dental pulp cells	Increased alkaline phosphatase activity in human dental pulp cells
Viapiana et al. [88]	ZrO_2_ (nanosize is not reported) * Portland cement	30	-Setting time -compressive strength -flow -film thickness -radiopacity -solubility -dimensional stability -formaldehyde release	Film thickness requires further reduction
Li et al. [89]	ZrO_2_ (50–75 nm) * Portland cement	20	-hydration chemistry	Biocompatibility not compromised. Accelerated hydration
**Dental Composites**				
Ilie et al. [90]	GO-ZrO_2_ HA-ZrO_2_ (10–40 nm)	0.3 GO-ZrO_2_ 15 HA-ZrO_2_	-light transmittance -flexural strength, modulus, Weibull parameters -plastic and elastic deformation parameters	-Improved mechanical properties -Optical properties require adjustments
Hesaraki et al. [91]	3-YSZ (≤100 nm)	5, 10	-flexural strength compressive strength	-Increase in mechanical strength
Wu et al. [92]	ZrO_2_ coated with Zr (OH)_4_ (50 nm)	10	-flexural strength -elastic modulus -Weibull analysis	-Improvement of mechanical properties
Dai et al. [30]	ZrO_2_ coated with Zr (OH)_4_ (50 nm)	2.5, 5, 7.5	-flexural strength -translucency	-5% wt presented the highest strength
Furman et al. [93]	Zirconium propoxide (<100 nm)	10, 20, 30	-flexural strength -fracture toughness	-Reduced flexural strength
**Dental Adhesives**				
Provenzi et al. [94]	ZrO_2_ (<25 nm)	0.5, 1, 4.8, 9.1	-degree of conversion -radiopacity -tensile bond strength softening in solvent	1 wt% led to a significantly higher degree of conversion
Martins et al. [95]	ZrO_2_ (20–30 nm)	15, 25, 30, 50	-micro-hardness radiopacity	Increased micro-hardness and radiopacity
Lohbauer et al. [32]	YSZ (20–50 nm)	5, 10, 15, 20	-micro-tensile -bond strength	Increasing concentration led to higher bond strength values

* = Type of cement in which the nanoparticles were incorporated.

## Data Availability

Data is contained within the article.
